# Cerebellar Cysticercosis Caused by Larval *Taenia crassiceps* Tapeworm in Immunocompetent Woman, Germany

**DOI:** 10.3201/eid1912.130284

**Published:** 2013-12

**Authors:** Vasileios Ntoukas, Dennis Tappe, Daniel Pfütze, Michaela Simon, Thomas Holzmann

**Affiliations:** Krankenhaus der Barmherzigen Brüder, Regensburg, Germany (V. Ntoukas, D. Pfütze);; University of Würzburg, Würzburg, Germany (D. Tappe);; University of Regensburg, Germany (M. Simon, T. Holzmann)

**Keywords:** Taenia crassiceps, cysticercosis, molecular identification, PCR, zoonosis, cerebellar infection, central nervous system, immunocompetent, tapeworm, parasites, Germany

## Abstract

Human cysticercosis caused by *Taenia crassiceps* tapeworm larvae involves the muscles and subcutis mostly in immunocompromised patients and the eye in immunocompetent persons. We report a successfully treated cerebellar infection in an immunocompetent woman. We developed serologic tests, and the parasite was identified by histologic examination and 12s rDNA PCR and sequencing.

*Taenia crassiceps* tapeworms are intestinal parasites of carnivores (final hosts), mostly foxes and dogs, in North America, Europe, and Russia. Rodents are natural intermediate hosts that harbor the cyst-like larvae (metacestodes, cysticerci) in their body cavities or subcutaneously, where the larvae proliferate by asexual budding (*1*). Prevalence among foxes in Germany and Lithuania is high (*2*), 24% and 26.4%, respectively. In contrast, prevalence in Denmark is low, only 0.2% (*3*). Although humans rarely serve as intermediate hosts, an increasing number of zoonotic infections have emerged in recent years (*1*,*4*–*12*). Infection of humans is thought to occur after consumption of food or water contaminated with infective ova shed in carnivore feces (*1*). All recognized cases involving the muscles or subcutis of humans have been associated with underlying immunosuppression (*1*,*4*–*7*,*12*), except for 1 case (Ronald Neafie, pers. comm). In contrast, intraocular infections (*8*–*10*) do not seem to require an impaired immune system (Table).

**Table:** Cases of *Taenia crassiceps* tapeworm infection in humans*

Patient residence	Site of infection	Type of immunosuppression	Reference
Germany	Cerebellum	None	This article
Switzerland	Subcutis and muscle, upper limb	AIDS	(*4*)
Germany	Subcutis and muscle, forearm and hand	NHL	(*1*)
United States (Oregon)	Subcutis, shoulder	None	Ronald Neafie, pers. comm.
United States (Maine)	Eye (subretinal)	None	Ronald Neafie, pers. comm.)
France	Subcutis and muscle, arm	AIDS	(*7*)
France	Subcutis and muscle, forearm	AIDS	(*5*)
USA (Missouri)	Eye (subretinal)	None	(*9*)
France	Subcutis and muscle	AIDS	(*12*)
Austria	Eye (anterior chamber)	None	(*8*)
Germany	Subcutis and muscle, back	AIDS	(*6*)
Canada (Ontario)	Eye (anterior chamber)	None	(*10*)

*NHL, non-Hodgkin lymphoma.

We describe a case of intracranial *T*. *crassiceps* tapeworm cysticercosis with severe involvement of the cerebellum. Combined surgical removal of the larvae and treatment with albendazole and praziquantel led to a complete cure in this nonimmunocompromised patient. The organism was unequivocally identified by molecular methods, thus avoiding a misdiagnosis of *Taenia solium* tapeworm cysticercosis.

## The Study

**Figure 1:**
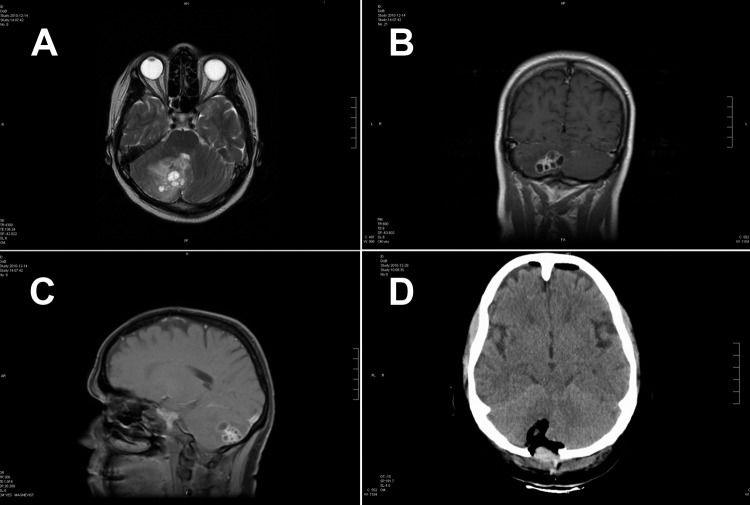
Magnetic resonance (MR) and computed tomographic images of the brain of a 51-year-old woman infected with *Taenia crassiceps* tapeworm larvae, Germany. A) Transverse view, T1-weighted MR image. The 30 × 30 mm parasitic lesion with perifocal edema is located in the right hemisphere of the cerebellum and caused ataxia, headache, and nausea. The fourth ventricle is compressed. B) Coronal view, T2-weighted MR image. The cyst-like appearance of the parasitic tissue is clearly visible. This lesion can be misinterpreted as cerebral echinococcosis, racemose cysticercosis caused by a *Taenia solium* tapeworm, or coenurosis. C) Sagittal view, MR image with contrast enhancing agent. D) Transverse view, computed tomographic image after surgery. No residual parasitic masses, only the parenchymal defect in the cerebellum after resection of *T*. *crassiceps* tapeworm larvae, are visible.

In 2011, in Regensburg, southern Germany, a 51-year-old German woman was hospitalized because of progressive headache, nausea, and vomiting. The signs and symptoms had started 2 weeks before, and intensity had been increasing ever since. At the time of admission, the patient showed cerebellar ataxia but no further neurologic deficits. She did not have fever or other symptoms. She had no known chronic preconditions or recent hospital stays and had never taken immunosuppressant drugs. She had no family history of neurologic symptoms or malignant diseases. Cranial computed tomography was performed and demonstrated a tumorous lesion (≈30 × 30 mm) in the right cerebellar hemisphere compressing the fourth ventricle. Magnetic resonance imaging revealed a multicystic mass with little perifocal edema (Figure 1). The patient’s leukocyte count was elevated (27.4 × 10^9^ cells), and a differential count indicated 84% neutrophils, 8% lymphocytes, and 4% eosinophils. Aspartate aminotransferase (129 U/L), alanine aminotransferase (335 U/L), and gamma glutamyl transferase (196 U/L) levels were elevated, and total plasma protein concentration was slightly lowered (4.7 g/dL). Kidney function test results, C-reactive protein levels, and gamma globulin levels were within normal limits. 

Craniotomy revealed subdural and intracerebellar jelly-like tumorous tissue. The tumor, which consisted of multiple spherical masses with diameters of 2–4 mm, was resected. No infiltration of meningeal structures or the skull was evident.

**Figure 2:**
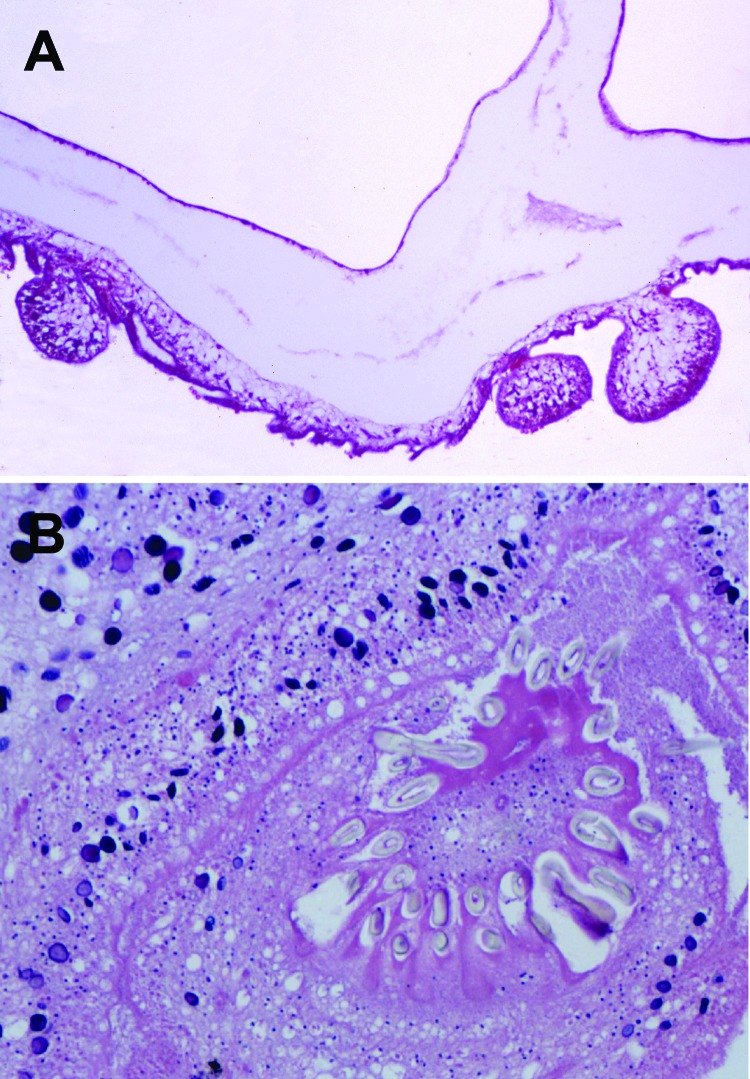
Histologic section through *Taenia crassiceps* tapeworm larvae removed from the cerebellum of a 51-year-old woman, Germany. A) Section through parasite body showing multiple connected bladders (asexual buddings) at the caudal end. Original magnification ×20. B) Transverse section through the parasite’s protoscolex showing numerous hooklets, similar to *T*. *solium* tapeworm larvae. Original magnification ×40. Like the *Taenia* *solium* tapeworm that causes cysticercosis, and in contrast to different tapeworms that cause coenurosis (*T.* [*Multiceps*] *multiceps*, *T.* [*Multiceps] serialis*) or echinococcosis, the *T*. *crassiceps* tapeworm has only 1 invaginated protoscolex, but it is on a very long neck (*Cysticercus longicollis*). The hooklets of *T. crassiceps* tapeworms are larger than those of *T. solium* tapeworms and have a larger blade length than handle length. The small hooklets measure 123 µm; the large hooklets measure 167 µm.

Because an intracranial parasitosis or tumor was suspected, serum, tissue, and fluid from the cystic lesion were examined. Gross and histologic aspects of the excised tissue revealed typical structures for cestode larvae (Figure 2). Serum and tissue samples were sent to a reference laboratory for further examination. Serologic test results for echinococcosis, which used crude and recombinant antigen ELISAs, and indirect hemagglutination test results were negative (*11*). Commercial Western blots for cysticercosis and echinococcosis (LDBIO Diagnostics, Lyon, France) showed weak atypical bands of ≈47 kDa and 30 kDa, respectively (Technical Appendix Figure 1). For the tissue samples, cestode-specific PCRs selective for the parasite’s mitochondrial 12S rRNA gene (*13*) and mitochondrial cytochrome c oxidase subunit I gene (*14*) were positive. After sequencing and conducting a BLAST search (www.ncbi.nlm.nih.gov/blast/Blast.cgi) of the 380-bp and 450-bp amplicons, we found that the sequences showed 99% and 100% homology with the *T. crassiceps* tapeworm, respectively. 

A crude *T*. *crassiceps* ELISA similar to an in-house *Echinococcus multilocularis* assay was set up (*11*) by using laboratory-kept *T*. *crassiceps* tapeworm larvae from another human patient (*1*). Serum samples from 10 healthy blood donors served as negative controls, and a standardized threshold index of 1.0 was calculated. Because no serum from patients with proven *T*. *crassiceps* tapeworm infections was available to use as a positive control, we used serum from patients with histologically confirmed cystic echinococcosis (5 patients), alveolar echinococcosis (7 patients), and peripheral cysticercosis (2 patients). All serum samples were positive, showing indices of 1.2–9.1, 1.4–6.6, and 2.2–3.3, respectively. The patient’s serum, however, had an index below the threshold (0.76). When 5-µm cryosections from *T*. *crassiceps* tapeworm larvae were used for immunofluorescence tests, the patient’s serum exhibited a weak tegumental signal (Technical Appendix Figure 2).

After surgery, the patient was given praziquantel (600 mg twice daily) and albendazole (400 mg twice daily) as described (*1*) for 3 months. The postoperative course was uneventful, the patient recovered rapidly, and there were no clinical or radiographic signs of recurrence after a follow-up period of 18 months. Extended imaging investigations showed no further sites of infection. 

When the patient was asked about potential risk factors, she indicated that she had been living with her dog near a forest in a local rural area for many years. Consumption of wild berries or mushrooms possibly contaminated by fox feces could not be excluded. The dog, which had not regularly undergone deworming, had access to the garden and the nearby forest. 

## Conclusions

In recent years, more reports of human infection with *T*. *crassiceps* tapeworms have surfaced, possibly because of increasing numbers of immunocompromised persons. The patient described here showed no evidence of an impaired immune system, and the parasite was found in an immunologically privileged site. Similarly, for patients with *T*. *crassiceps* larvae infection of the eye and for patients with neurocysticercosis caused by *T. solium*, immunosuppression does not seem to be a prerequisite for infection. *T*. *crassiceps* tapeworm larvae are apparently able to infect the same variety of human tissues as *T*. *solium*, but do so much more rarely. Most infections, including the case reported here, have been reported from southern Germany (*1**,**6*) and France (*5*,*7*,*12*). Other infections of humans have been reported from neighboring Switzerland and Austria and from North America. Similar to the distribution of alveolar echinococcosis in Europe (another larval cestode disease for which the red fox is also the final host), a contiguous area with microfoci of transmission could hypothetically be possible. Diagnosis depends on the radiographic image resembling a racemose cysticercus (because of the multicystic aspect of *T*. *crassiceps* tapeworm infections) and correct identification of the parasite by gross morphologic and histologic appearance by experienced pathologists or by molecular techniques. 12S rDNA PCR proved to be a useful tool that is not widely used (*13*,*14*), and its use helped avoid the misdiagnosis of *T. solium* tapeworm neurocysticercosis. 

The diagnosis of *T*. *crassiceps* tapeworm infection is demanding for laboratories because no tests are commercially available. As described here, unusual serologic reactions displayed on tests for other larval helminthoses should raise the level of suspicion for a different causative agent. Of note, the serologic diagnosis of neurocysticercosis caused by *T*. *solium* can be difficult; commercial tests showed sensitivity <72% (*15*). Such a low sensitivity could hypothetically explain the negative ELISA result for the patient reported here, for whom no peripheral tissues were infected, in contrast to the control serum used. Thus, the true prevalence of human disease caused by *T*. *crassiceps* tapeworms could be underestimated, and future seroprevalence studies using ELISA and immunofluorescence testing can possibly shed more light on this type of infection. The source of infection for this patient remains unclear, but her dog is probably the major risk factor (*1*,*8*,*10*). As a preventive measure, carnivorous pets should undergo regular deworming.

Technical Appendix Cysticercosis and echinococcosis Western blot patterns of the serum from the patient with *Taenia* *crassiceps* infection, and immunofluorescence tests with cryosections of *T. crassiceps* larvae.
