# Supplementary material for: Cerebellar Cysticercosis Caused by Larval Taenia crassiceps Tapeworm in Immunocompetent Woman, Germany
Source: Emerg Infect Dis. 2013 Dec;19(12):2008–11. doi: 10.3201/eid1912.130284 (PMC3840866; doi:10.3201/eid1912.130284)
Supplement: Technical Appendix — Cysticercosis and echinococcosis Western blot patterns of the serum from the patient with Taenia crassiceps infection, and immunofluorescence tests with cryosections of T. crassiceps larvae. [file 13-0284-Techapp-s1.pdf]

Article DOI: http://dx.doi.org/10.3201/eid1912.130284

Cerebellar Cysticercosis Caused by Larval *Taenia crassiceps* Tapeworm in Immunocompetent Woman, Germany

Technical Appendix

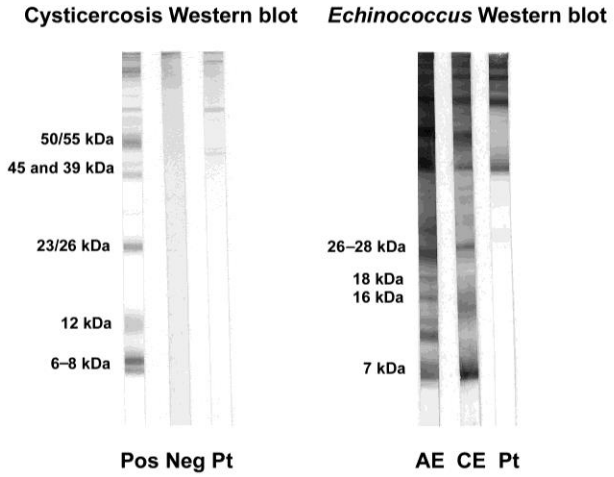

Technical Appendix Figure 1. Cysticercosis and echinococcosis Western blot patterns of the serum from the patient with *Taenia*. *crassiceps* tapeworm infection. Weak atypical bands are seen at ≈47 kDa (cysticercosis blot) and 30 kDa (echinococcosis blot). Pos, positive control serum of a patient with cysticercosis. Neg, negative control serum; Pt, serum from patient reported in this article; AE, alveolar echinococcosis pattern; CE, cystic echinococcosis pattern.

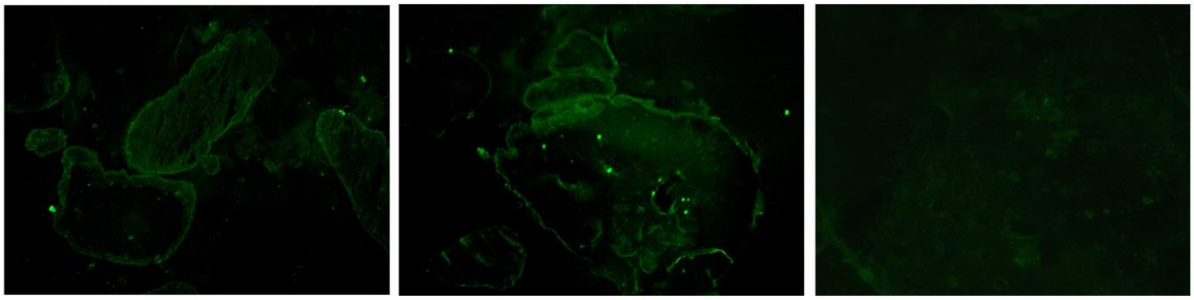

Technical Appendix Figure 2. Immunofluorescence images of cryosections of *Taenia crassiceps* tapeworm larvae. Left, serum from the patient with cerebellar *T*. *crassiceps* tapeworm infection. This serum had an index in the *T*. *crassiceps* ELISA of 0.76 (negative). Weak tegumental fluorescence is

visible. Middle, cross-reacting serum from a patient with peripheral cysticercosis. This serum had an ELISA index of 3.2 (positive). Tegumental fluorescence is visible. Right, negative control serum from a patient with negative serologic test results for echinococcosis and cysticercosis. This serum had an ELISA index of 0.14 (negative). Only background fluorescence is seen. All serum samples were used in a dilution of 1:100, secondary goat antibody against human IgG was conjugated with fluorescein isothiocyanate (1:400, Fluoline G, bioMérieux, Marcy l'Etoile, France). Original magnification ×200.
